# Dietary patterns in mild cognitive impairment and dementia in older adults from Yucatan, Mexico

**DOI:** 10.3389/fnut.2024.1335979

**Published:** 2024-05-15

**Authors:** Angel Gabriel Garrido-Dzib, Berenice Palacios-González, María Luisa Ávila-Escalante, Erandi Bravo-Armenta, Azalia Avila-Nava, Ana Ligia Gutiérrez-Solis

**Affiliations:** ^1^Hospital Regional de Alta Especialidad de la Península de Yucatán, IMSS-Bienestar, Mérida, Yucatán, Mexico; ^2^Facultad de Medicina, Universidad Autónoma de Yucatán, Mérida, Yucatán, Mexico; ^3^Laboratorio de Envejecimiento Saludable del Instituto Nacional de Medicina Genómica (INMEGEN), Centro de Investigación sobre el Envejecimiento, Ciudad de México, Mexico

**Keywords:** dementia, mild cognitive impairment, older adults, nutrition, dietary pattern, Mexico

## Abstract

**Background:**

Some dietary patterns and dietary components have an important role in preventing and helping to improve patients’ quality of life of individuals with Mild Cognitive Impairment (MCI) and dementia. In Mexico, it is unknown what the dietary patterns are among older adults with MCI and dementia. We aimed to identify the dietary patterns of older adults with MCI and dementia living in Yucatan, Mexico.

**Methods:**

A cross-sectional study was carried out among 39 patients as controls and 34 individuals as cases (MCI and dementia). A food frequency questionnaire collected diet information, anthropometric and clinical parameters, and lifestyle characteristics. The dietary patterns were evaluated through Partial Least-Squares Discriminant Analysis (PLS-DA).

**Results:**

The food groups that showed discrimination between groups and were classified into the dietary patterns of MCI and dementia individuals were “pastries and cookies,” “soups,” and “legumes.” The dietary pattern of older adults without cognitive impairment was characterized by “nuts and seeds,” “candies,” “vegetables,” “coffee and tea,” and “water.” The consumption of “pastries and cookies” showed an increasing correlation with serum insulin levels (*r* = 0.36, *p* = 0.01), and “soups” showed an inverse correlation with total cholesterol levels (*r* = −0.36, *p* = 0.02) in patients with MCI and dementia. In controls, there is a positive correlation between the consumption of “nuts and seeds” (*r* = 0.333, *p* = 0.01) and “vegetables” (*r* = 0.32, *p* = 0.02) with levels of urea; “coffee and tea” showed a positive association with levels of insulin (*r* = 0.378, *p* = 0.05).

**Conclusion:**

The dietary pattern of individuals with MCI and dementia has some nutritional deficiencies. Including an adequate intake of vegetables, fruits, and protein could improve the quality of life of subjects living with these conditions in Yucatan, Mexico.

## 1 Introduction

The 2022 census shows that around 15 million older adults live in Mexico. Currently, 14% of the Mexican population is 60 or older (1). Older age remains one of the main risk factors associated with mild cognitive impairment (MCI) and dementia (2, 3). The Study on Aging and Dementia in Mexico (SADEM) reported a prevalence of 7.8% of older adults with Alzheimer’s Disease (AD) living in Mexico; this is the most common type of dementia among older people (4). MCI is a neurocognitive state between normal cognitive aging and dementia and can be detected in younger adults around 55 years old (5). Both conditions are characterized by a deterioration of cognitive function that prevents daily activities and reduces the quality of life (6).

Even though the physiopathology of dementia is still under research, some well-established lifestyle factors, such as physical activity, reducing alcohol consumption, and having an adequate nutritional status, have been associated with reducing the risk of MCI and dementia (7–9). Some dietary and nutritional components have been linked to the deterioration or improvement of cognitive function (10).

A recently published meta-analysis found that the diet of individuals with MCI and dementia living in Latin American Countries is characterized by a lower intake of fruits and vegetables and high consumption of simple carbohydrates and animal protein (11). As mentioned above, the importance of diet in delaying the intrinsic causes of pathologies and diseases associated with aging has been recognized for a long time. Over the last decades, evidence has accumulated on the protective role of bioactive compounds on the risk of chronic non-communicable diseases and even longevity and aging. However, understanding the net impact of diet on health is more complex than studying isolated components or foods. Humans do not consume individual foods but mixed food combinations that form a dietary pattern. Therefore, from a physiological point of view, analyzing eating habits considering the interactions between different foods and their components is of primary interest (12). As a result, it’s crucial to evaluate the diet as a whole. The principal components analysis (PCA) considers complex diets and multiple food groups instead of individual nutrients, specific foods, or food groups for pattern classification. Besides, dietary patterns can reflect an individual’s food preferences; therefore, dietary pattern analysis may add more information to reflect the complexity of dietary intake and provide new insights into the whole foods diet (13).

More recently, it has been suggested that dietary patterns and some dietary components have an important role in preventing these conditions and in helping to improve patients’ quality of life during the disease at different stages (12, 14). Many healthy dietary patterns have been associated with improved cognitive function, and these dietary patterns have several components in common: a high consumption of fruits, vegetables, and whole grains, along with a low consumption of red meat and sweets. However, it is unknown what the dietary patterns are among older adults with MCI and dementia in Mexico; therefore, this study aimed to identify the dietary patterns of older adults with MCI and dementia living in Yucatan, Mexico.

## 2 Methods

### 2.1 Study design

The present cross-sectional study was carried out between February 2022 and January 2023. The group of cases included individuals who attended the outpatient specialty unit of neurology and geriatrics at the Regional High Speciality Hospital, IMSS-Bienestar in Merida, Yucatan, Mexico, with diagnoses of MCI and dementia. For controls, individuals without MCI or dementia were included. Patients under medical treatment for specific diseases in the cardiology, endocrinology, respiratory, urology, and oncology units and individuals with implanted medical devices such as pacemakers or prostheses were excluded from the study. To ensure the representativeness of the population, a sample size was calculated using an unmatched case–control formula for an unknown population with a 95% two-sided confidence level, 90% power, 50% of controls exposed, and a 0.06 odds ratio (15). From this calculation, the minimum required sample size was 24 individuals in each group. Finally, a sample of 39 patients as controls and 34 individuals as cases (MCI and dementia) were selected for this study.

### 2.2 Ethical clearance

The study was approved by the Ethics Committee of the Regional High Speciality Hospital, IMSS-Bienestar (no. CONBIOETICA-31-CEI-002-20170731) in connection with a research project (identification code: 2021–012), following the guidelines for human experiments as laid down in the Helsinki Declaration. The participants signed the informed consent form before the start of the study.

### 2.3 Mild cognitive impairment and dementia assessment

Participants had a previously established diagnosis of dementia or MCI by a neurologist or geriatrician. Diagnoses were made following internationally accepted criteria for dementia. The diagnostic and statistical manual of mental disorders-V (DSM-V), ICD-10, and MCI Petersen criteria were used (16, 17). Additionally, subjects with dementia were evaluated using the mini-mental state examination (MMSE) and MCI individuals by the Montreal Cognitive Assessment (MoCA) (18, 19).

### 2.4 Dietary assessment

The habitual diet was assessed using a semi-quantitative food frequency questionnaire (SFFQ) previously validated in the Mexican population and administered by a trained dietician (20, 21); if the participant could not respond, the information was provided by the primary caregiver. The SFFQ contained 140 items grouped into previously pre-validated food groups by Gaona et al. (22) (Supplementary Table S1). For the analysis, only those food groups that reported 70% or more of consumption were included; 19 food groups were obtained. The SFFQ data were then converted to grams or milliliters per week consumption by multiplying the standard serving size. Foods were recorded in grams (g), and drinks and broths in milliliters (mL). However, to have similar units of measurement among foods and beverages, the milliliters per week were converted to cup units by dividing between 240 mL, resulting in cups per week.

### 2.5 Anthropometric parameters, blood pressure, and lifestyle characteristics

Waist circumference (WC) (cm) was measured from individuals to the nearest 0.1 cm using an anthropometric tape measure (Lufkin, United States) at the midpoint between the lower costal margin and the superior of the iliac crest. Weight and height were measured using a calibrated scale. Body mass index (BMI) was calculated as weight divided by the square of height (kg/m^2^). Blood pressure (BP) (systolic and diastolic, SBP and DBP, respectively) was estimated after 15 min of rest in the sitting position using an automatic electronic sphygmomanometer (Omron, Japan). A pre-validated questionnaire was used to collect a brief clinical history of the participants, such as pre-existing diseases and lifestyle.

### 2.6 Serum biomarkers

Following standard procedure, a trained researcher collected a blood sample after 12 h of overnight fasting. Clinical biochemistry tests were done following standard protocol to estimate levels of triglycerides (TG) (mg/dL), high-density lipoprotein cholesterol (HDL-C) (mg/dL), total cholesterol (mg/dL), fasting plasma glucose (FPG) (mg/dL), insulin (μIU/mL), glycated hemoglobin (HbA1c) (%), urea (mg/dL), creatinine (mg/dL), and uric acid (mg/dL). A pre-validated equipment (autoanalyzer COBAS^®^ Integra 400 Plus, Roche Diagnostics) was used for the clinical biochemistry tests.

### 2.7 Statistical analysis

The statistical packages Jamovi (version 2.25, Sydney, Australia) and SPSS version 15.00 (IBM Corp, Armonk, New York, United States) were used to analyze the data. Descriptive statistics were calculated for the control and cases (MCI and dementia) groups. The Shapiro–Wilk test was used to check the normality. Clinical and serum biomarkers characteristics were presented as means ± standard deviation (SD) or medians and interquartile range (IQR). Proportions and corresponding percentages (%) were reported for comorbidities and lifestyles and tested by *Pearson’s chi*-*squared test*. Continuous variables between groups were compared using the independent *t*-test or the Mann–Whitney U test. A Spearman correlation analysis assessed the relationship between group foods and serum biomarkers. For all analyses, statistical significance was set at *p* < 0.05.

The web-based platform Metaboanalyst 5.0 was used to identify discrimination among food groups through partial least-squares discriminant analysis (PLS-DA) (23). For dietary patterns, the selected methods were: Row-wise; normalization: Normalization to constant sum; Data transformation: Cubic Root Transformation; and Data scaling: Autoscaling. Permutation testing was conducted to minimize the possibility that the observed separation on PLS-DA was by chance. Additionally, for cross-validation (R2, Q2, and accuracy), model validation was performed using a 2000 times permutation test. A loading scatter plot was constructed to determine the variables discriminating between the groups. The variable importance in the projection (VIP) plot was performed based on their significance in discerning studies from both groups. VIP cutoff >1.0 was designated since the number of variables in this study was less than 100 (24).

## 3 Results

### 3.1 Clinical characteristics of the study population

Seventy-three participants were analyzed, including 39 controls and 34 cases (17 individuals with MCI and 17 with dementia). Overall, cases (73.4 ± 10.10) were older than controls (67.2 ± 6.52) (*p* = 0.002), had a higher frequency of women (68.4%, *p* = 0.097), hypertension (50%, *p* = 0.056), and metabolic syndrome (MetS) (70%, *p* = 0.913). Besides, taking medication was significantly higher among participants with MCI and dementia (91%, *p* < 0.0001) (Table 1).

**Table 1:** General characteristics among controls and cases.

Characteristics	Controls *n* = 39	95% CI	Cases *n* = 34	95% CI	*p*-value
Age (Years)	67.2 ± 6.52	(65.2–69.3)	73.4 ± 10.1	(70.4–76.8)	**0.002**
Male, *n* (%)	9 (23)		14 (41)		0.097
WC (cm)	92.6 ± 10.6	(89.3–96)	93.8 ± 12.0	(89.8–97.9)	0.659
BMI (kg/m^2^)	22.73 ± 4.48	(27.7–30.5)	26.9 ± 5.05	(26.1–29.7)	0.315
SBP (mmHg)	129 ± 14.9	(125–134)	132 ± 22.2	(124–129)	0.584
DBP (mmHg)	80.0 (72.0–85.0)	(75.1–81.6)	72.0 (68.3–79.8)	(70.6–80.3)	0.065
Comorbidities, *n* (%)					
T2D	9 (23)		13 (38)		0.159
Hypertension	11 (28)		17 (50)		0.056
MetS	20 (51)		17 (50)		0.913
Life style					
Physical activity (min/week)	90.0 (0–240)	(92.6–226)	65.0 (0–180)	(73.2–216)	0.590
Alcohol consumers, *n* (%)	11 (28)		5 (15)		0.164
Pharmacotherapy, *n* (%)	22 (56)		31 (91)		**0.0001**

Data is presented using mean ± standard deviation or median (Interquartile range). Differences between groups were evaluated with independent *t*-test or Mann–Whitney test. *p*-value less than 0.05 was considered statistically significant. 95% CI, 95% confidence intervals; WC, waist circumference; BMI, body mass index; SBP, systolic blood pressure; DBP, diastolic blood pressure; T2D, type 2 diabetes; MetS, metabolic syndrome. The bold values are statistically significant.

Levels of urea (39.1 (29.1–44.5) vs. 31.8 (23.3–34) mg/dL, *p* = 0.013), creatinine (0.94 (0.80–1.06) vs. 0.73 (0.64–0.88) mg/dL, *p* < 0.001), and percentage of HbA1c (5.96 (5.64–6.51) vs. 5.61 (5.30–6.03) %, *p* = 0.033) were significantly higher among cases than controls. Overall levels of other parameters such as triglycerides, cholesterol, FPG, insulin, and UA increased in the case group (Table 2).

**Table 2:** Biochemical parameters among controls and cases.

Characteristics	Controls *n* = 39	95% CI	Cases *n* = 34	95% CI	*p*-value
Triglycerides (mg/dL)	122 (86.5–166)	115–171	139 (101–197)	130–176	0.246
HDL-C (mg/dL)	49.5 (45.5–59.1)	48.9–63.8	46.5 (40.3–55.4)	45.7–59.1	0.134
Cholesterol (mg/dL)	183 ± 34.9	172–194	186 ± 47.5	170–202	0.707
FPG (mg/dL)	102 (89.0–116)	99.6–121	104 (97.1–119)	103–116	0.266
Insulin (μIU/mL)	8.79 (5.80–17.6)	8.80–14.1	7.63 (0.20–12.4)	5.85–15.6	0.151
HbA1c (%)	5.61 (5.30–6.03)	5.64–6.35	5.96 (5.64–6.51)	5.90–6.46	**0.033**
Urea (mg/dL)	31.8 (23.3–34)	26.7–37	39.1 (29.1–44.5)	31.8–44.4	**0.013**
Creatinine (mg/dL)	0.73 (0.64–0.88)	0.71–0.86	0.945 (0.80–1.06)	0.84–1.41	**<0.001**
UA (mg/dL)	4.79 ± 2.70	4.41–5.17	5.31 ± 2.40	4.74–5.87	0.132
CRP (mg/dL)	3.20 (1.30–6.25)	2.92–7.48	2.40 (1.20–4.65)	0–19.1	0.442

Data is presented using mean ± standard deviation or median (Interquartile range). Differences between groups were evaluated with independent *t*-test or Mann–Whitney test. *p*-value less than 0.05 was considered statistically significant. 95% CI, 95% confidence intervals; HDL-C, high density lipoprotein cholesterol; FPG, fasting plasma glucose; HbA1c, glycated hemoglobin; UA, acid uric; CRP, C-Reactive protein. The bold values are statistically significant.

### 3.2 Dietary patterns according to neurocognitive disease

**Figure 1:**
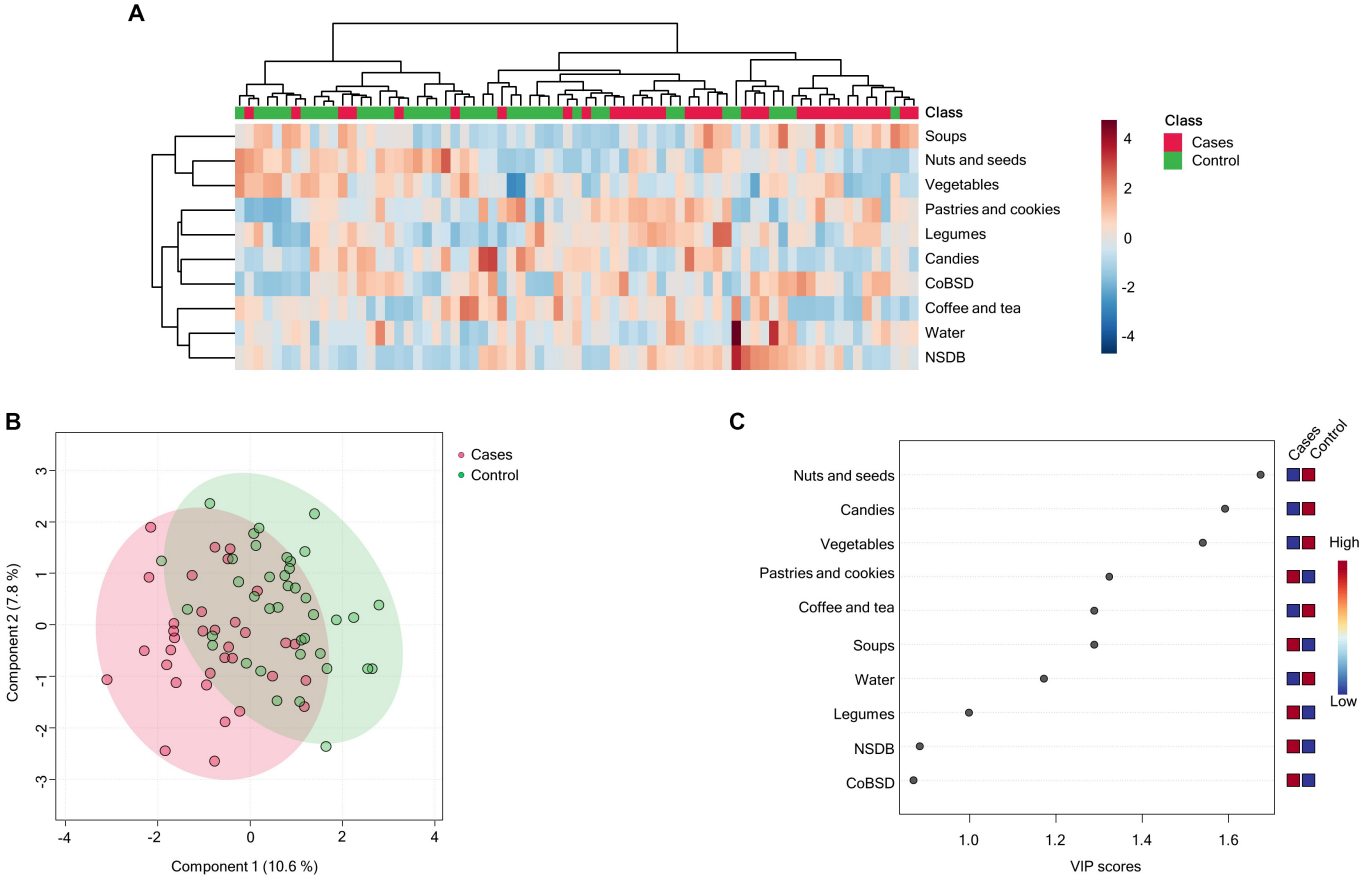
Dietary patterns are profiled according to the group of cases (MCI and dementia) and control. **(A)** Hierarchical heatmap for dietary patterns: control (green) and cases (red); red and blue show increasing and decreasing food group consumption, respectively. **(B)** The PLS-DA plot shows separation between cases (red) and controls (green). The explained variances are shown in brackets (accuracy: 0.69; R2: 0.354; Q2: 0.108; permutation *p* value <0.005). **(C)** The VIP analysis represents the relative contribution of food groups to the variance among groups. A high VIP score indicates a greater contribution of the food group to the dietary pattern. Red and blue boxes on the right indicate whether food group consumption is increased (red) or decreased (blue).

The PLS-DA score plots presented slight evidence of separation according to having MCI and dementia vs. older adults without neurocognitive disease (Figures 1A,B). This difference between groups showed an accuracy of 0.69, R2: 0.354, Q2: 0.108, and permutation *p* value <0.005. The VIP plot revealed that “pastries and cookies,” “soups,” and “legumes” are responsible for discrimination among patients with MCI and dementia (Figure 1C). “Nuts and seeds,” “candies,” “vegetables,” “coffee and tea,” and “water” were the food groups that were found to be in charge of differentiation in the control group.

### 3.3 Correlations between serum biomarkers and food groups in the study population

**Figure 2:**
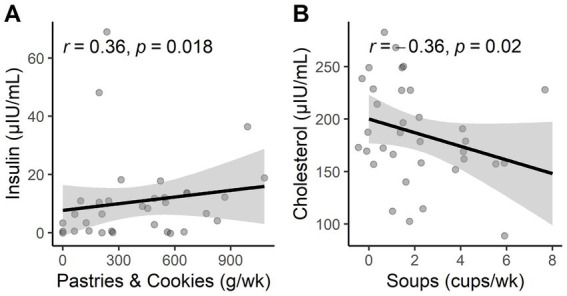
**(A)** Statistical correlations of pastries and cookie consumption with insulin levels in cases. **(B)** Statistical correlations of soup consumption with cholesterol levels in cases.

**Figure 3:**
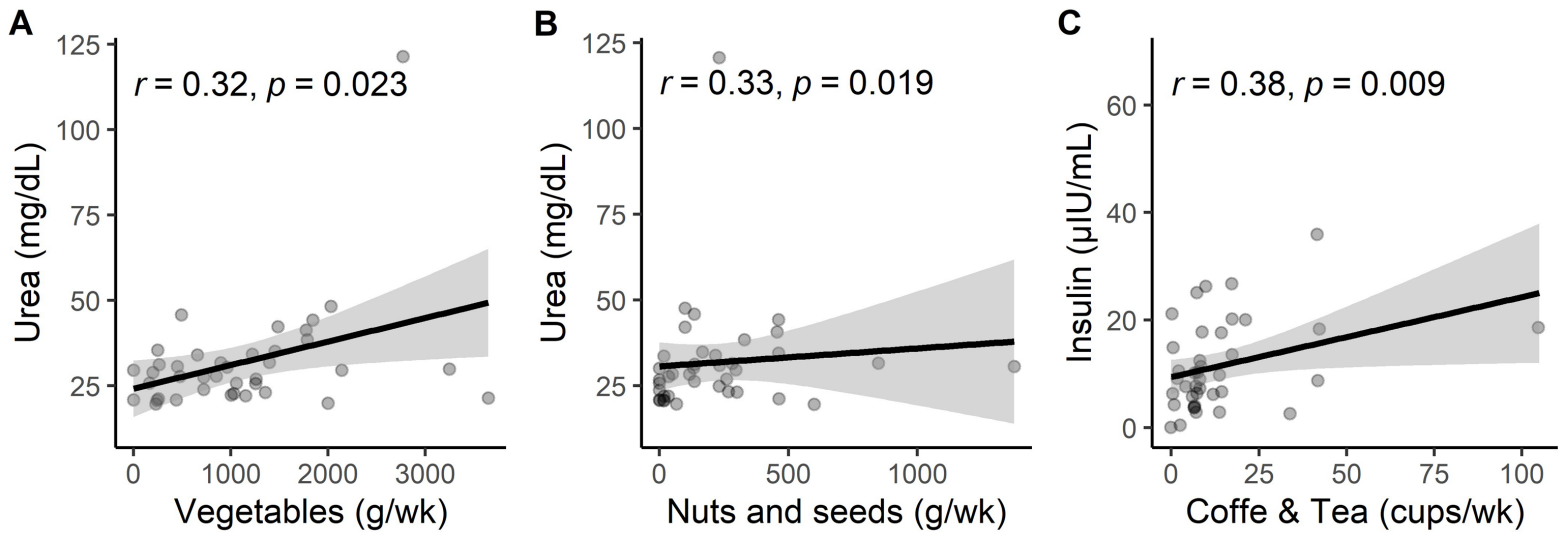
**(A)** Statistical correlations of vegetable consumption with urea levels in the control group. **(B)** Statistical correlations of nuts and seeds consumption with urea levels in the control group. **(C)** Statistical correlations of coffee and tea consumption with insulin levels in the control group.

The consumption of “pastries and cookies” showed an increasing correlation with serum insulin levels (*r* = 0.36, *p* = 0.018), and the consumption of “soups” showed an inverse correlation with total cholesterol levels (*r* = −0.36, *p* = 0.02) in patients with MCI and dementia (Figures 2A,B; Supplementary Table S2). In controls, there is a positive correlation between the consumption of “nuts and seeds” (*r* = 0.333, *p* = 0.018) and “vegetables” (*r* = 0.32, *p* = 0.023) with levels of urea, meaning levels of urea significantly increase as the consumption of nuts and seeds and vegetables increases (Figures 3A,B). Additionally, the consumption of “coffee and tea” showed a positive association with levels of insulin (*r* = 0.38, *p* = 0.009) (Figure 3C; Supplementary Table S3).

The correlation between serum biomarkers and food groups, adjusting sex and age, was performed through a linear regression analysis using a rank transformation of variables. In the case group, the interaction of insulin and “pastries and cookies” was strong and significant (β = 0.391, *p* = 0.026); however, the correlation between “soups” and cholesterol levels was no longer significant after adjusting (β = −0.35, *p* = 0.052). In the control group, the interaction between insulin levels and “coffee and tea” (β = 0.33, *p* = 0.033), urea with “nuts and seeds” (β = 0.37, *p* = 0.021), and urea and “vegetables” (β = 0.35, *p* = 0.025) stayed significant and stronger after adjusting for sex and age.

## 4 Discussion

This study identified two dietary patterns in the Mexican older adult population. The food groups that showed discrimination between groups and were classified into the dietary patterns of MCI and dementia individuals were “pastries and cookies,” “soups,” and “legumes.” The dietary pattern of older adults without cognitive impairment was characterized by the following food groups: “nuts and seeds,” “candies,” “vegetables,” “coffee and tea,” and “water.”

The focus of scientific nutrition research has shifted from examining the impact of individual foods and nutrients on health to examining dietary patterns that represent the combined intake of several meals and nutrients (13, 25). However, it is important to consider that dietary patterns might differ based on factors like age, culture, way of life, socioeconomic situation, and state of health (26).

In our study, the MCI and dementia dietary pattern included both “*healthy”* and “*unhealthy”* food groups, which are characterized by high consumption of “pastries and cookies,” “soups,” and “legumes,” this was also found among Korean and Chinese adults with MCI (27, 28). The food group “pastries and cookies” has been distinguished by its high content of added sugars, saturated and trans fats, salt, and additives that have been associated with cognitive impairment in prior research (29). The consumption of these products has been linked to intestinal dysbiosis, the promotion of proinflammatory cytokines, and metabolic alterations, which generate alterations in various organs, including brain damage (30–32). On the other hand, the food group “legumes” was primarily characterized by the consumption of beans, which have conflicting findings regarding their impact on health. Since ancient times, eating beans has been a staple of the Mexican diet (33). Flavonoids are the principal compounds of beans and have been associated with a lower risk of AD (34); however, there is still a gap in our knowledge of the underlying mechanisms and their association with brain health. Moreover, there is no daily intake recommendation for flavonoids in the diet.

Mixed vegetables and chicken soup were the types of food that were more frequent in the food group “soups.” Poultry and vegetables (especially leafy green vegetables) are two foods recommended in the diet to lower or prevent dementia (21). However, there is not sufficient literature that examines the benefits of the consumption of mixed vegetables and chicken soup on individuals with dementia. Mixed vegetables and chicken soup comprise several ingredients that could work synergistically at multiple targets on a neurocognitive level (35, 36).

The dietary pattern in the control group of older adults included healthier food groups in comparison to the MCI and dementia dietary patterns. Food groups such as “nuts and seeds,” “vegetables,” “coffee and tea,” and “water” were reported. Only “candies” could be identified as an unhealthy food group.

The reported more frequent vegetables among the study population included tomatoes and onions. One of the main bioactive compounds of the tomato is lycopene (37). Lycopene is a natural neuroprotective agent. This carotenoid seems to contribute to cognitive longevity and treat several neuronal diseases, including cerebral ischemia, Parkinson’s disease (PD), AD, and depression (38). Quercetin is a bioactive compound of the onion and another type of flavonoid (39). Its cognitive function was examined in a randomized, double-blind, placebo-controlled, parallel-group comparative clinical trial that evaluated the consumption of quercetin-rich onion for 24 weeks compared to quercetin-free onion as a placebo. Quercetin-rich onions reduce age-related cognitive decline, possibly by improving emotional conditions in healthy older adults from Japan (40). The pretreatment with quercetin significantly enhanced the expression levels of endogenous antioxidant enzymes such as Cu/Zn superoxide dismutase (Cu/Zn, SOD), manganese SOD (Mn/SOD), catalase (CAT), and GSH peroxidase in the hippocampal CA1 pyramidal neurons of animals suffering from ischemic injury. Thus, quercetin may be a neuroprotective agent for transient ischemia (41).

Caffeine is an alkaloid found in coffee, tea, and soft drinks (42). Consumption has been reported to decrease the risk of dementia (43). Vincenzo et al. found that people who regularly drank one to two cups of coffee daily had a reduced incidence of MCI than those who did not drink coffee (44). However, some other studies have shown that caffeine intake does not help improve neurocognitive abilities in men, only in women (45).

A high frequency of sugar consumption is associated with several conditions in older adults, such as type 2 diabetes, stroke, cancer, and dementia. Excessive sugar consumption has been linked to neurocognitive dysfunction. Also, it was found in animal models using rats that high sugar consumption may cause neuroinflammation in the hippocampal region (46).

Although the number of participants was fewer than the variables, PLS-DA has been reported to be able to use small sample sizes (47). In our analysis, the model fit (R2: 0.354) was moderate, and consistency (Q2: 0.108) showed that the results obtained had an accuracy of 0.69. Additionally, the permutation (10/2000) was reported to be statistically significant (*p* value <0.005). These results suggest that the obtained dietary patterns were relatively consistent and reliable; however, a larger sample could result in a stronger association with more power.

Some associations were found between serum biomarkers, food groups, and dietary patterns. Among the dietary patterns in cases (MCI and dementia individuals) where the consumption of “pastries and cookies” was positively associated with insulin levels, eating sugar-filled pastries or candies can cause blood glucose levels to rise, leading the pancreas to produce the hormone insulin (48). High levels of insulin and glucose will cause damage to the blood vessels of the brain, leading to a decline in mental capacity (49). Another food group that showed a significant association with cholesterol levels was “soups.” A negative relationship was found between “soups” and cholesterol, meaning that levels of cholesterol decrease with increasing consumption of “soup,” this could be explained by the addition of vegetables to soup, which increases the consumption of healthy foods such as fiber (50) and phytosterols (51) in the diet, both of which can help lower cholesterol levels.

In the dietary pattern of the control group, it was reported that there was a positive association between increased levels of urea and the consumption of “nuts and seeds” and “vegetables. “It has been reported that intake of these food groups could reduce urea levels in adults. However, these contradictory findings could be explained by the addition of salt to both food groups. Typically, the preparation and consumption of these food groups in this region of Mexico are accompanied by high amounts of salt (52). Lastly, insulin levels were positively associated with the consumption of coffee and tea; this association could be explained by the fact that 59% of older adults from this study reported sweetening their coffee and tea. In particular, the contribution of each source of salt and sugar consumption can vary according to culture, age groups, habits, and dietary practices.

Moreover, the dietary assessment tools need to allow for precise measurement. Further studies may focus on identifying dietary patterns and the intake of micro and macronutrients among MCI and dementia populations. These days, “Omics” technologies have advanced our understanding of individual and population health at the systems level. Specifically, metabolomics has enabled the identification of “metabotypes,” easily measured in urine or blood. Therefore, this method could result in objective biomarkers of foods, nutrients, and dietary patterns in individuals with MCI and dementia.

### 4.1 Limitations

The absence of information on the participants’ type of dementia is a limitation of the current work; this will be crucial to investigate because Yucatán has been reported to have high rates of obesity, overweight, and other comorbidities linked to MCI and dementia. The results, however, provide the first information about the type of dietary pattern in older Mexican adults with these conditions. The implementation of long-term studies will help to identify and comprehend the association and underlying mechanisms of dietary patterns and serum biomarkers.

## 5 Conclusion

We found that the MCI and dementia dietary patterns have some nutritional deficiencies. They were characterized by high consumption of “pastries and cookies,” “soups,” and “legumes.” An adequate intake of vegetables, fruits, and protein could improve the quality of life of subjects living with these conditions in Yucatan.

## Data availability statement

The original contributions presented in the study are included in the article/Supplementary material, further inquiries can be directed to the corresponding authors.

## Ethics statement

The studies involving humans were approved by Comité de Investigación & Comité de Ética en Investigación del Hospital Regional de Alta Especialidad. The studies were conducted in accordance with the local legislation and institutional requirements. The participants provided their written informed consent to participate in this study.

## Author contributions

AG-D: Data curation, Investigation, Methodology, Project administration, Validation, Writing – review & editing, Visualization. BP-G: Investigation, Methodology, Supervision, Validation, Visualization, Writing – original draft, Writing – review & editing, Software. MÁ-E: Investigation, Resources, Supervision, Validation, Writing – review & editing. EB-A: Investigation, Resources, Supervision, Writing – review & editing. AA-N: Investigation, Resources, Supervision, Validation, Visualization, Writing – review & editing. AG-S: Conceptualization, Investigation, Methodology, Project administration, Resources, Supervision, Validation, Writing – original draft, Writing – review & editing.
