# Corrigendum: Dietary patterns in mild cognitive impairment and dementia in older adults from Yucatan, Mexico

**DOI:** 10.3389/fnut.2024.1514921

**Published:** 2024-12-04

**Authors:** Angel Gabriel Garrido-Dzib, Berenice Palacios-González, María Luisa Ávila-Escalante, Erandi Bravo-Armenta, Azalia Avila-Nava, Ana Ligia Gutiérrez-Solis

**Affiliations:** ^1^Hospital Regional de Alta Especialidad de la Península de Yucatán, IMSS-Bienestar, Mérida, Yucatán, Mexico; ^2^Facultad de Medicina, Universidad Autónoma de Yucatán, Mérida, Yucatán, Mexico; ^3^Laboratorio de Envejecimiento Saludable del Instituto Nacional de Medicina Genómica (INMEGEN), Centro de Investigación sobre el Envejecimiento, Ciudad de México, Mexico

**Keywords:** dementia, mild cognitive impairment, older adults, nutrition, dietary pattern, Mexico

In the published article, there was an error in the **Acknowledgment statement**. In the current Acknowledgment statement, a sentence was missing acknowledging the Secretaría de Educación, Ciencia, Tecnología e Innovación de la Ciudad de México. The old statement read as:

“The authors are very grateful to the participants. We also thank Julio Vega for helping with data analysis and Valeria Magallón and Geovanni Chávez for additional technical support. AG-D received a scholarship from CONAHCyT (CVU 1142884).”

The correct statement should have been written as:

“The authors are very grateful to the participants. We also thank Julio Vega for helping with data analysis and Valeria Magallón and Geovanni Chávez for additional technical support. AG-D received a scholarship from CONAHCyT (CVU 1142884). The present work was carried out under the framework of the Secretaría de Educación, Ciencia, Tecnología e Innovación de la Ciudad de México (SECTEI/227/2021).”

The authors apologize for this error and state that this does not change the scientific conclusions of the article in any way. The original article has been updated.

