# Supplementary material for: Dietary patterns in mild cognitive impairment and dementia in older adults from Yucatan, Mexico
Source: Front Nutr. 2024 May 15;11:1335979. doi: 10.3389/fnut.2024.1335979 (PMC11334730; doi:10.3389/fnut.2024.1335979)
Supplement: Supplementary file 1 [file Table_1.DOCX]

**SUPPLEMENTARY MATERIAL**

**Supplementary Table S1.** Food components for each food groups that were used for dietary patterns.

| Food group | Foods and beverages included |
| --- | --- |
| Sugar-sweetened beverages | Milk with added sugars, drinkable yoghurt (natural, fruit-added, light) and Mexican beverages (atoles with or without milk), soft drinks, industrialized fruit juices and nectars, and sugar-sweetened natural fruit water, probiotic drink (Yakult®). |
| Non-sugar-sweetened dairy beverages | Milk without added sugars (whole, semi-skim, skim, lactose-free, powder milk) |
| Non-sugar-sweetened non-dairy beverages | Non-sugar-sweetened natural fruit, light soft drinks, and other low-calorie beverages. |
| Fruits | Strawberry, cranberries, prune, jicama, papaya, pineapple, pomelo, grape, orange, mandarin orange, lemon, apple, pear, cantaloupe, watermelon, peach, banana, guava, and mango. |
| Vegetables | Cucumber, carrot, tomato, chayote, chard, spinach, cabbage, lettuce, broccoli, corn, zucchini, chili pepper, onion, vegetable pear, cauliflower, green bean, nopal, canned or frozen vegetables. |
| Dairy products (non-beverage) | Yoghurt (natural, fruit-added, light) and cheese (cottage, panela, chihuahua or type-manchego, type-gouda) |
| Legumes | Beans, lentils, chickpeas, broad beans, homemade pot or refried beans, industrialized pot or refried beans. |
| Cereal-based salty dishes | Rice, pasta, and instant soups. |
| Corn-based salty dishes | Mexican pozole and tamales, or Mexican street food: non-fried without meat, non-fried with meat, fried without meat or fried with meat (*quesadillas, enchiladas, tacos, tlacoyos, gorditas*). |
| Fast food | Hamburgers, pizza, hot dogs, and sandwiches |
| Eggs | Boiled eggs, scramble eggs, fried eggs, and egg-battered vegetables |
| Meat, poultry, and fish | Poultry, beef, pork, fish, and shellfish |
| Processed meats | Processed ham and sausage (pork or turkey), spicy pork sausage. |
| Pastries and cookies | Donuts, cakes, pies, cupcakes, cookies, and pastries |
| Desserts | Fried plantains, gelatins, flan, ice cream, frozen juice bars, sorbet, and Danonino® (petit Suisse cheese). |
| Candies | Refined sugar, solid or powder chocolate, candies, marshmallows, lollipops, and ketchup. |
| Nuts and seeds | Peanuts, nuts, almonds, pumpkin seeds, and avocado. |
| Added fats | Butter, lard, vegetable oil, mayonnaise, cream, coffee creamer, vegetable cream, popcorn, and chips. |
| Tortillas | Corn tortilla (homemade or commercially prepared). The corn tortilla is a staple food in traditional. Mexican cooking: it is a thin, circular unleavened flatbread made of corn flour |
| Soups | Soups and broths (beef, chicken, and vegetable) |
| Ready-to-eat cereals | Ready-to-eat cereals (all kinds): basic, light, fiber-added, multi-ingredient, sugar-sweetened or flavored |
| Breads | Bread, White bread, whole-wheat bread, crackers, wheat tortilla |
| Coffee and tea | Coffee and tea without sugar |
| Alcoholic beverages | Alcoholic beverages |
| Water | Water |

Note: A bold font implies that food groups that were reported as having 70% of consumption among the study population.

**Supplementary Table S2.** Correlations among food groups and clinical parameters in MCI and dementia patients.

|  | **Legumes** | **Pastries and cookies** | **Soups** |
| --- | --- | --- | --- |
| Triglycerides (mg/dL) | 0.043 | 0.333 | -0.009 |
| HDL-C (mg/dL) | -0.023 | -0127 | -0.198 |
| Cholesterol (mg/dL) | 0.153 | 0.037 | -0.355* |
| FPG (mg/dL) | 0.205 | -0.038 | 0.104 |
| Insulin (µIU/mL) | 0.309 | 0.362** | 0.052 |
| HbA1c (%) | 0.031 | -0.147 | 0.053 |
| Urea (mg/dL) | -0.030 | -0.112 | 0.013 |
| Creatinine (mg/dL) | -0.304 | 0.134 | 0.187 |
| UA (mg/dL) | -0.057 | 0.137 | 0.072 |
| CRP (mg/dL) | 0.072 | 0.043 | 0.033 |

HDL-C: High density lipoprotein cholesterol; FPG: Fasting plasma glucose; HbA1c: Glycated hemoglobin; UA: Acid uric; CRP: C-Reactive Protein. *Note.* * p < 0.05, ** p < 0.01, *** p < 0.001

**Supplementary Table S3.** Correlations among food groups and clinical parameters in older adults.

|  | **Nuts and seed** | **Vegetables** | **Candies** | **Water** | **Coffee and tea** |
| --- | --- | --- | --- | --- | --- |
| Triglycerides (mg/dL) | -0.231 | -0.260 | 0.002 | 0.108 | 0.075 |
| HDL-C (mg/dL) | 0.184 | 0.191 | -0.082 | 0.006 | -0.305 |
| Cholesterol (mg/dL) | -0.197 | -0.200 | 0.185 | 0.019 | -0.138 |
| FPG (mg/dL) | 0.264 | 0.005 | -0.034 | 0.181 | 0.014 |
| Insulin (µIU/mL) | 0.010 | -0.038 | -0.067 | 0.156 | 0.378* |
| HbA1c (%) | 0.127 | -0.032 | -0.247 | 0.275 | 0.063 |
| Urea (mg/dL) | 0.333** | 0.321** | -0.189 | 0.186 | -0.180 |
| Creatinine (mg/dL) | 0.185 | 0.172 | -0.070 | 0.208 | -0.141 |
| UA (mg/dL) | 0.082 | 0.068 | 0.124 | 0.186 | 0.121 |
| CRP (mg/dL) | 0.111 | 0.108 | -0.008 | 0.046 | 0.008 |

HDL-C: High density lipoprotein cholesterol; FPG: Fasting plasma glucose; HbA1c: Glycated hemoglobin; UA: Acid uric; CRP: C-Reactive Protein. *Note.* * p < 0.05, ** p < 0.01, *** p < 0.001
